# Supplementary material for: Neonatal Consumption of Oligosaccharides Greatly Increases L-Cell Density without Significant Consequence for Adult Eating Behavior
Source: Nutrients. 2019 Aug 21;11(9):1967. doi: 10.3390/nu11091967 (PMC6769936; doi:10.3390/nu11091967)
Supplement: Supplementary file 1 [file nutrients-11-01967-s001.pdf]

**Supplementary data**

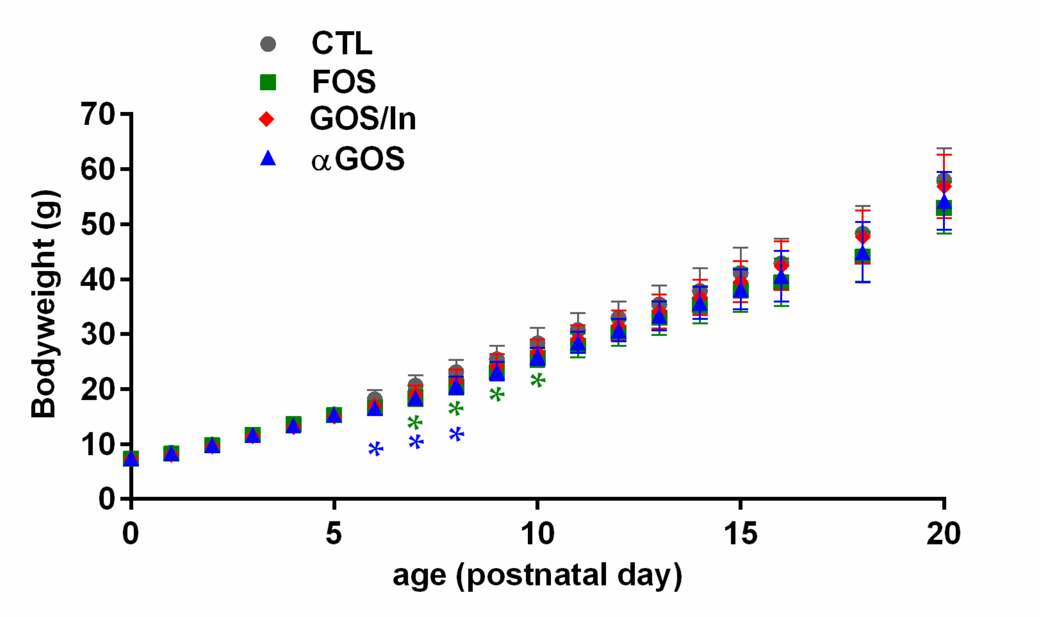


**Figure S1.** Postnatal growth of suckling rats in the different groups of OS supplementation. Data are means +/- SD collected from the total effective of rats (*n* = 15–16 per group from PND0 to PND14 then *n* = 8 per group). Asterisks indicate significant difference compared with CTL, their color refers to the treatment received between PND 5-14/15.

**Table S1.** Primer sequences.

| Target | Forward Primer | Reverse Primer | Accession Number |
|---|---|---|---|
| *β–actin* | CTATCGGCAATGAGCGGTTCC | GCACTGTGTTGGCATAGAGGTC | NM_031144 |
| *Atoh1* | TGTTAGCAACG TGTGACTTC | CAGACCAGAGACAGAGAT ACG | NM_001109238 |
| *Gcg* | CTAATGCTGGTACAAGGCAG | GTGAATGTGCCCTGTGAATG | NM_012707 |
| *Neurod1* | CACGCAGAAGGCAAGGTGTC | TGGTCATGTTTCCACTTCCTGT | NM_019218 |
| *Foxa1* | GTTCCGCACAGGGTTGGATA | CTGACCGGGACAGAGGAGTA | NM_012742 |
| *Neurog3* | Not available | Not available | NM_021700 |
| *Pax4* | CCCAAGGGTATTGGGGGAAG | GGATACACTGGGAGCCTTGTC | NM_031799 |
| *Pax6* | ATACCTACACCCCTCCGCAC | TGAGTCCTGTTGAAGTGGTTCC | NM_013001 |
| *PYY* | AGCGGTATGGGAAAAGAGAAGTC | ACCACTGGTCCACACCTTCTG | NM_001034080 |

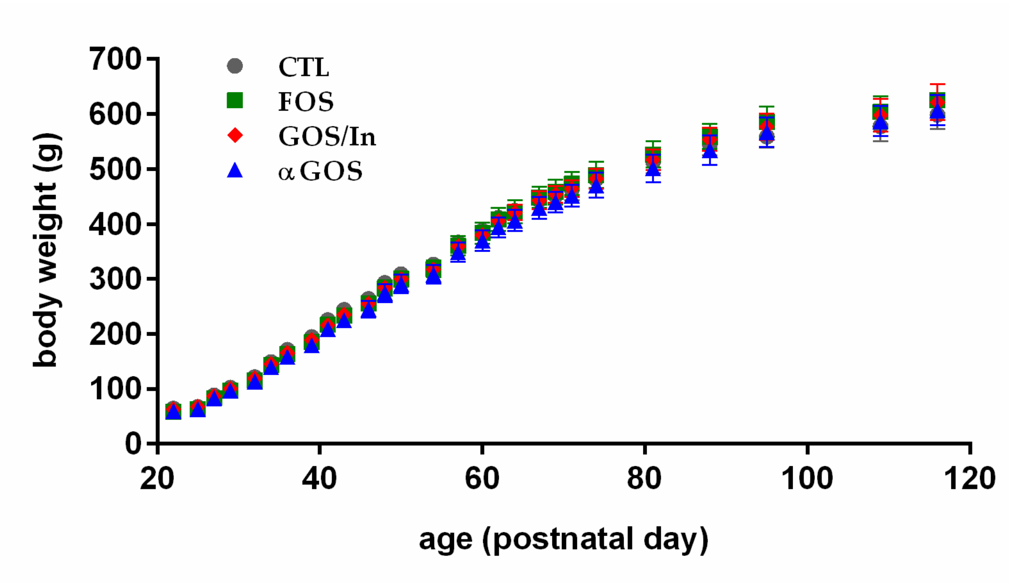


**Figure S2.** Growth of weaned rats until adulthood. Data are means +/− SD (*n* = 8 per group).

**Table S2.** Concentration of SCFA in ileal and cecal contents at PND 124/126.

| | Ileal Concentrations (mM) | | | Ceco-Colonic Concentrations (mM) | | | |
|---|---|---|---|---|---|---|---|
| **Treatment** | **Acetate** | **Propionate** | **Butyrate** | **Acetate** | **Propionate** | **Butyrate** | **Minors** |
| CTL | 2.1 ± 1.0 | 0.1 ± 0.1 | ND [1] | 13.5 ± 3.3 | 3.5 ± 0.8 | 2.7 ± 1.1 | 1.4 ± 0.5 |
| FOS | 2.2 ± 1.6 | 0.1 ± 0.1 | ND | 11.1 ± 1.9 | 2.9 ± 0.5 | 2.1 ±0.7 | 1.2 ± 0.2 |
| GOS/In | 2.7 ± 1.5 | 0.2 ± 0.2 | ND | 11.7 ± 3.1 | 3.3 ± 0.9 | 1.9 ± 0.7 | 1.3 ± 0.5 |
| αGOS | 2.9 ± 1.4 | 0.1 ± 0.1 | ND | 10.7 ± 1.9 | 2.9 ± 0.5 | 1.7 ± 0.5 | 1.2 ± 0.3 |

[1.] ND, non-detectable. Data are means ± SD (*n* = 8 per groups).

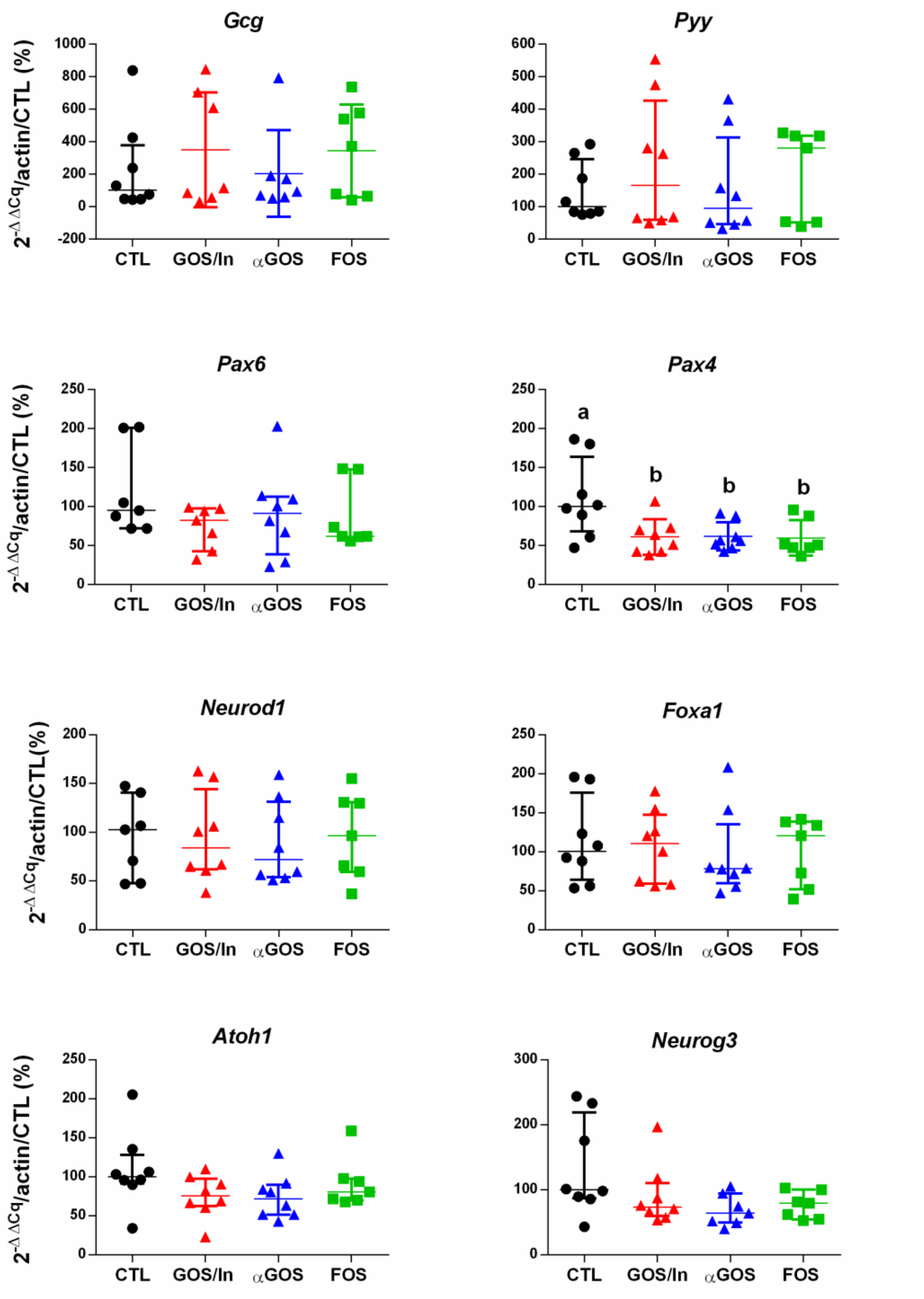


**Figure S3.** Relative expression of gene implied in the endocrine lineage and in L-cells differentiation in the colon. Different letters indicate significant difference between groups ($p < 0.05$). Data are fold-change expressed in % of CTL group. Individual values, median with interquartile range are plotted ($n = 7$ to 8 per group).

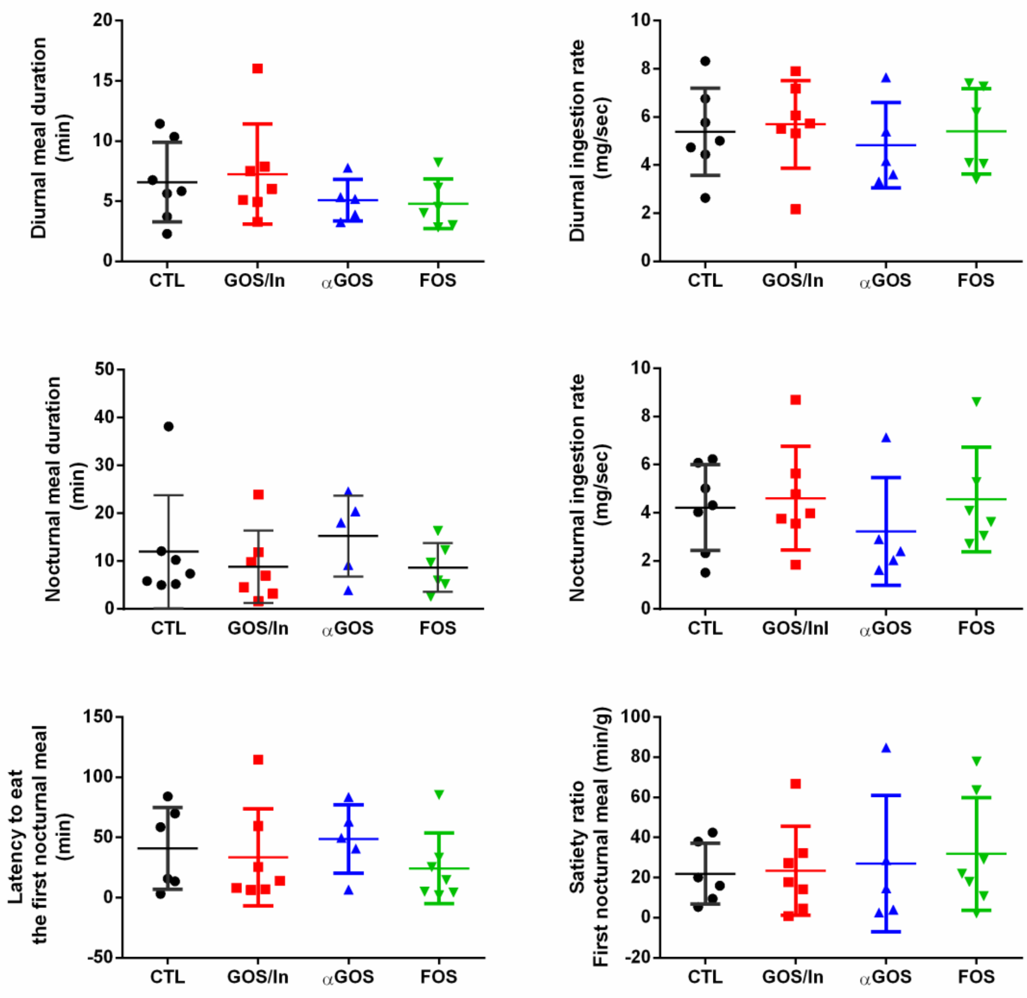


**Figure S4.** Supplemental parameters of meal pattern: meal duration and ingestion rate (mean of total meals per phase) during the diurnal and nocturnal phases. Latency to eat and satiety ratio are represented for the first meal at the beginning of the nocturnal phase. Individuals, means and SD are plotted (*n* = 5 to 7 per group).

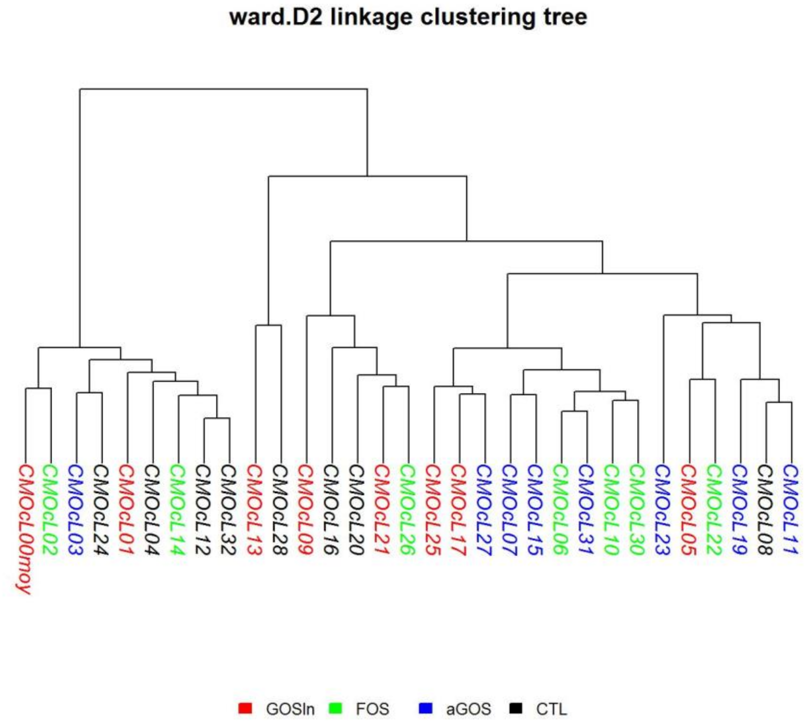


**Figure S5.** Hierarchical clustering of phylogenetically informed distance matrix computed using the unweighted UniFrac metric for cecal contents collected at PND 124/126.
